# A method for cryopreservation and single nucleus RNA-sequencing of normal adult human interventricular septum heart tissue reveals cellular diversity and function

**DOI:** 10.1186/s12920-021-01011-z

**Published:** 2021-06-15

**Authors:** Amy Larson, Michael T. Chin

**Affiliations:** Molecular Cardiology Research Institute, Tufts Medical Center, 800 Washington Street, Box 80, Boston, MA 02111 USA

**Keywords:** Myocardial biology, Cardiovascular disease, Functional genomics, Gene expression and regulation, Human heart tissue, Single nucleus RNA-sequencing

## Abstract

**Background:**

Single cell sequencing of human heart tissue is technically challenging and methods to cryopreserve heart tissue for obtaining single cell information have not been standardized. Studies published to date have used varying methods to preserve and process human heart tissue, and have generated interesting datasets, but development of a biobanking standard has not yet been achieved. Heart transcription patterns are known to be regionally diverse, and there are few single cell datasets for normal human heart tissue.

**Methods:**

Using pig tissue, we developed a rigorous and reproducible method for tissue mincing and cryopreservation that allowed recovery of high quality single nuclei RNA. We subsequently tested this protocol on normal human heart tissue obtained from organ donors and were able to recover high quality nuclei for generation of single nuclei RNA-seq datasets, using a commercially available platform from 10× Genomics. We analyzed these datasets using standard software packages such as CellRanger and Seurat.

**Results:**

Human heart tissue preserved with our method consistently yielded nuclear RNA with RNA Integrity Numbers of greater than 8.5. We demonstrate the utility of this method for single nuclei RNA-sequencing of the normal human interventricular septum and delineating its cellular diversity. The human IVS showed unexpected diversity with detection of 23 distinct cell clusters that were subsequently categorized into different cell types. Cardiomyocytes and fibroblasts were the most commonly identified cell types and could be further subdivided into 5 different cardiomyocyte subtypes and 6 different fibroblast subtypes that differed by gene expression patterns. Ingenuity Pathway analysis of these gene expression patterns suggested functional diversity in these cell subtypes.

**Conclusions:**

Here we report a simple technical method for cryopreservation and subsequent nuclear isolation of human interventricular septum tissue that can be done with common laboratory equipment. We show how this method can be used to generate single nuclei transcriptomic datasets that rival those already published by larger groups in terms of cell diversity and complexity and suggest that this simple method can provide guidance for biobanking of human myocardial tissue for complex genomic analysis.

**Supplementary Information:**

The online version contains supplementary material available at 10.1186/s12920-021-01011-z.

## Background

Heart disease is a leading cause of mortality in western society [1]. Understanding the complex interplay between molecules within cells and the interactions between cells in the healthy human adult heart is essential for developing novel treatment and improving current treatment strategies for various cardiac diseases. Identification of and characterization all cell types within normal human heart tissue is an important first step. Single cell RNA-sequencing (scRNA-seq) enables the study of cell heterogeneity within a tissue, and identification of small cell populations not detectable by bulk methods. Single cell transcriptomes provide insight into biological pathway activity, thereby illuminating biological function of the identified cell types.

Recent advances in commercialization of microfluidic systems for isolating individual cells and preparing single cell transcriptomic libraries have enabled researchers to study thousands of cells from a single tissue sample. Multiple studies analyzing the single cell transcriptional profiles in rodent embryonic and adult hearts have been reported, uncovering important cell populations, interactions and transcription patterns relevant to mammalian heart development and disease [2–5]. The adult human heart is particularly challenging, however, as fresh tissue is difficult to obtain due to scarcity of samples, and those that are obtained often must be frozen for future analysis. Procedures for harvesting and cryopreserving tissues for biobanking that facilitate advanced single cell analyses in the human heart have not yet been standardized. Porcine hearts have often been used to model human hearts for surgical and interventional procedures, given their comparable sizes [6]. Two recent studies surveyed multiple regions of the human heart through single nuclei RNA-sequencing (snRNA-seq), identifying differences in cell types and cell subtypes between the atria and ventricles, but also between the right and left ventricles [7, 8]. These studies used different tissue processing methods and involved large consortia for tissue collection, processing and data analysis. Only one of these studies included the interventricular septum (IVS) as one of the anatomical regions studied [8]. The IVS is an important region of the heart, playing roles in the function of both the left and right ventricle and is frequently abnormal in hypertrophic cardiomyopathy. Understanding the cell populations present in the normal adult IVS and their potential biological roles will provide insight into normal and diseased IVS function.

Here, we present a simple adult human heart tissue dissection and cryopreservation method that allows for later isolation of single nuclei for use in commercial microfluidic platforms to generate single nuclei-RNA sequencing libraries (Fig. 3a). This method can be performed by most labs with simple laboratory equipment and access to core facilities. This method of cryopreservation and processing is simpler than previously described methods and yields comparable results [7, 8]. Additionally, this method would be useful for tissues in which separation into single cells is challenging, such as the heart and brain. Using this method, we have generated a dataset demonstrating the cellular diversity of the normal adult human IVS.

## Methods

### Sample cryopreservation and RNA quality assessment

Fresh, discarded pig hearts were obtained from the Surgical Research Laboratory at Tufts Medical Center and immediately placed on ice. 100 mg samples were cut from the IVS, minced into 1 mm^3^ pieces and either cryostored or used immediately for nuclei isolation as described below. Human myectomy samples were minced and cryostored as described below. Unused donor hearts were perfused with Wisconsin solution and transported on ice. 100 mg samples were cut from the IVS, minced into 1 mm^3^ pieces, placed in 0.5 mL of CryoStor CS10 Freeze Media (STEMCELL Technologies), and stored in a MrFrosty (ThermoFisher) at 4 °C for 10 min and then transferred to − 80 °C overnight. Bulk RNA was isolated from a piece of tissue using the Qiagen RNeasy Plus Micro kit and then assessed on the Agilent Bioanalyzer 2100. Samples with an RNA Integrity Number greater than 8.5 were used in library preparation.

### Nuclei isolation, library preparation, and sequencing

Cryopreserved samples were thawed at 37 °C for 75 s and immediately placed on ice. In contrast with single cell dissociation methods that use harsh buffers, enzymatic treatments, and harsh mechanical separation which can result in stress-induced changes in the transcriptome, here nuclei were released from the fresh or frozen minced tissue via Dounce homogenization, a gentle dissociation method, using Triton X-100 to lyse the cell membrane and release the nuclei as previously described [9]. Homogenates were filtered through a Pluristrainer 10 µM cell strainer (Fisher Scientific) into a pre-chilled tube. Nuclei were pelleted by centrifuging at 500× *g* for 5 min at 4 °C. Nuclei pellets were washed and pelleted according to manufacturer protocol (10× Genomics). Nuclei were stained with trypan blue and counted on a hemocytometer to determine concentration prior to loading of the 10× Chromium device and samples were diluted to capture ~ 10,000 nuclei. Nuclei were separated into Gel Bead Emulsion droplets using the 10× Chromium device according to the manufacturers protocol (10× Genomics). Sequencing libraries were prepared using the Chromium Single Cell 3′ reagent V2 kit according to manufacturer’s protocol. Libraries were multiplexed (n = 4) and sequenced on a NovaSeq S2 (Illumina) to produce at least 50,000 reads per nucleus.

### Microscopy

Stained nuclei were visualized for counting purposes after trypan blue staining using a Nikon Eclipse Ti microscope with attached CoolSNAP EZ Camera and UV Source Lumencor light engine serial #1874 SOLA 6-LCR-SB, using Filter C169242 49028. Images were acquired using NIS-Elements BR Software, ver. 4.13 at 600× or 1000× magnification. No downstream processing was done, fields were not juxtaposed and no images were merged.

### Data processing

Sequencing reads were processed using Cell Ranger 3.0 [10], with reads mapping to the human reference genome GRCh38-1.2.0. The gene expression matrix was subset to only include reads from the nuclear genome, excluding mitochondrial genome reads. Quality control (QC) filtering, clustering, dimensionality reduction, visualization, and differential gene expression was performed using the development version of the R package Seurat in R 3.5.0. Each dataset was filtered so that genes that were expressed in three nuclei or more were included in the final dataset. The dataset was further sublet to exclude nuclei that had fewer than 200 genes expressed to remove droplets containing only ambient RNA, and to exclude nuclei with greater than 2000 genes to remove droplets that contained two nuclei. Datasets were individually normalized using Seurat’s SCTransform and then integrated using Seurat Integration standard workflow to reduce batch effects [11]. After QC filters, our final dataset included 24,858 nuclei, with an average 157,675 reads per nucleus.

Optimal clustering resolution was determined using Clustree [12] to identify the resolution where the number of clusters stays stable and was determined to be 0.9 for the integrated dataset. Differentially expressed genes in each cluster were identified using the “FindAllMarkers” function. Genes were only considered if they were expressed in 25% of the nuclei in that cluster with a log fold change threshold greater than 0.25.

### Cell type identification

Expression of known cell-specific markers were used to identify the major cell types. The list of differentially expressed genes for each cluster and their functions were used to identify clusters without an assigned identity, or to further refine the cell type. Additionally, the entire list of differentially expressed genes, both up- and down-regulated from the “FindAllMarkers” function (described above) were used for gene ontology analysis using GOstats [13] and pathway analysis using the Core Analysis in Qiagen’s Ingenuity Pathway Analysis software [14] were both used to further elucidate the identity and function of each cluster.

## Results

**Fig. 1:**
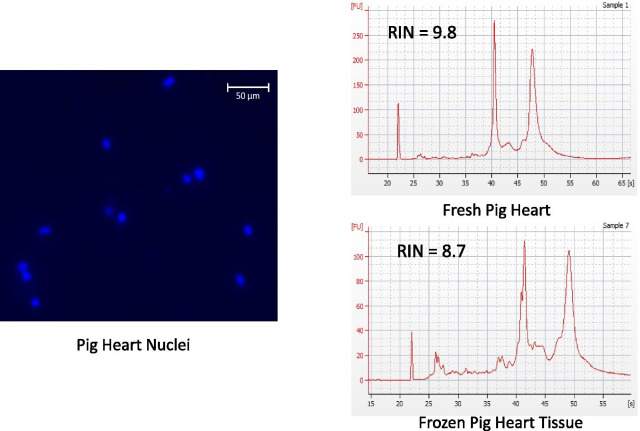
A pilot study of nuclei isolation and RNA quality from fresh and cryopreserved pig tissue. Discarded fresh pig heart tissue was minced and processed for nuclei isolation with representative nuclei shown in the left panel. A representative bioanalyzer tracing of RNA isolated from fresh pig heart tissue is shown in the upper right panel. A representative bioanalyzer tracing of RNA isolated from cryopreserved pig heart tissue is shown in the lower right panel

**Fig. 2:**
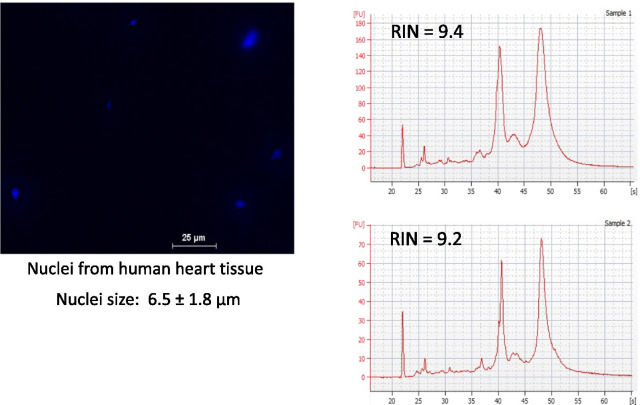
A pilot study of nuclei isolation and RNA quality from cryopreserved human myectomy tissue. Human myectomy tissue obtained from hypertrophic cardiomyopathy patients was minced and cryopreserved. Nuclei were prepared from cryopreserved tissue as shown in the left panel. Representative bioanalyzer tracings of RNA isolated from two different cryopreserved specimens are shown in the upper and lower right panels

**Fig. 3:**
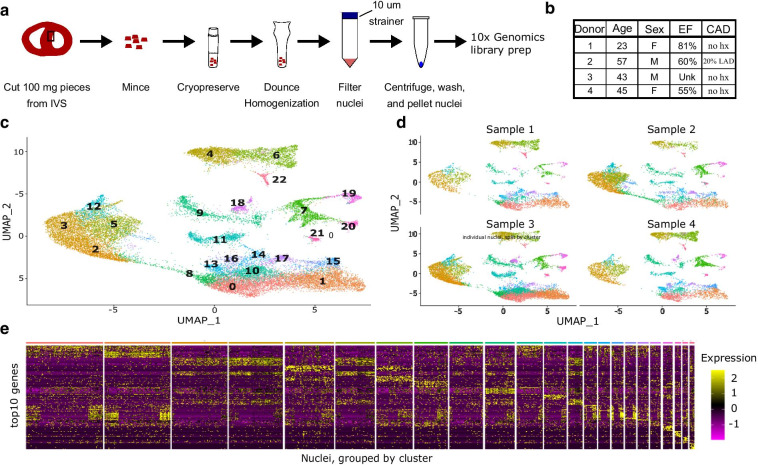
A method for cryopreserving and processing adult human heart tissue for single nuclei RNA-sequencing. **a** Workflow for cryopreserving adult human heart tissue and subsequent isolation and processing of nuclei for use in 10× Genomics microfluidics platform. **b** Demographics of the four unused donor hearts.**c**Batch-corrected Uniform Manifold Approximation and Projection (UMAP) of the integrated dataset. Each dot represents a single nucleus and is colored by cluster identity. Clusters are numbered in descending order of the number of nuclei assigned to that cluster. **d** Separated UMAP of each individual donor dataset demonstrating that every cell type was present in each donor and batch effects have been removed. **e** Heatmap of the top ten differentially expressed genes in each cluster across all the clusters. Each row is a different gene, colored by the expression level of that gene in each individual nucleus, with each column representing an individual nucleus. The expression of all of the top ten genes for each cluster across all clusters is displayed for each nucleus in the dataset. The nuclei are grouped by cluster, and the colored bar at the top indicates cluster number and corresponds to the colors in **c**

Pilot studies were performed on discarded pig hearts obtained from the surgical research laboratory. IVS tissue was dissected free, minced and either cryopreserved or immediately used for nuclei preparation. Nuclei could easily be isolated from either fresh or frozen tissue and RNA integrity remained high after cryopreservation (Fig. 1). We chose to isolate nuclei from heart tissue rather than whole cells, to reduce the expression of stress response genes brought on by harsh enzymatic or mechanical treatment [9] and to reduce challenges in microfluidic cell isolation since cardiomyocytes are much larger than other cell types within the heart. We then proceeded to use our cryopreservation protocol on human samples. We initially performed pilot studies on human myectomy tissue from patients with hypertrophic cardiomyopathy. Surgical samples obtained from the operating room were subject to our processing and cryopreservation protocol. Thawed tissue was then processed for nuclear isolation and bulk RNA analysis, demonstrating high quality RNA (Fig. 2). Human heart nuclei were measured in cross section and the diameter was found to average 6.5 ± 1.8 µm. Four unused donor hearts from both males and females between the ages 23–57, were obtained from New England Donor Services (Fig. 3b). The hearts were prescreened for evidence of heart disease, and any donors with a history of diabetes, hypertension, hyperlipidemia, smoking, rheumatoid arthritis, or any other potential contraindication were excluded from this study. Each heart was cut into transverse sections, and the samples from the IVS were weighed and minced, and then cryopreserved. Nuclei were released from the cryopreserved tissue using gentle homogenization (Fig. 3a). Each cryopreserved sample produced approximately half a million nuclei suitable for use in the 10× Genomic Single Cell Gene Expression system or the Fluidigm C1 system.

After sequencing and initial data processing with Cell Ranger software [10], each sample dataset was processed further to remove called nuclei that were likely droplets with only ambient RNA, or droplets that contained two nuclei. The four datasets were combined using the Seurat Integration function [11]. The final combined dataset included 24,858 nuclei. Overall clustering of the integrated dataset revealed 23 cell populations within the IVS, and this was visualized using the dimensionality reduction algorithm uniform manifold approximation and projection (UMAP, Fig. 3c), where each dot represents a single nucleus and is colored by cluster identity. Splitting the integrated dataset into the individual datasets reveals that all 23 clusters are present in each dataset and no single sample is driving the clustering (Fig. 3d). Differentially expressed genes for each cluster were determined using the Seurat function “FindAllMarkers”, and the full list of differentially expressed genes for each cluster is listed in Additional file 1: Table 1. The top ten differentially expressed genes from each cluster were determined and their expression levels in each single nucleus in the dataset were represented in a heatmap with nuclei grouped together by cluster identity across the x-axis (Fig. 3e). Analysis of the top ten differentially expressed genes in each cluster was sufficient to indicate cluster similarity (e.g. clusters 2 and 3), likely representing similar cell types with different biological functions.

**Fig. 4:**
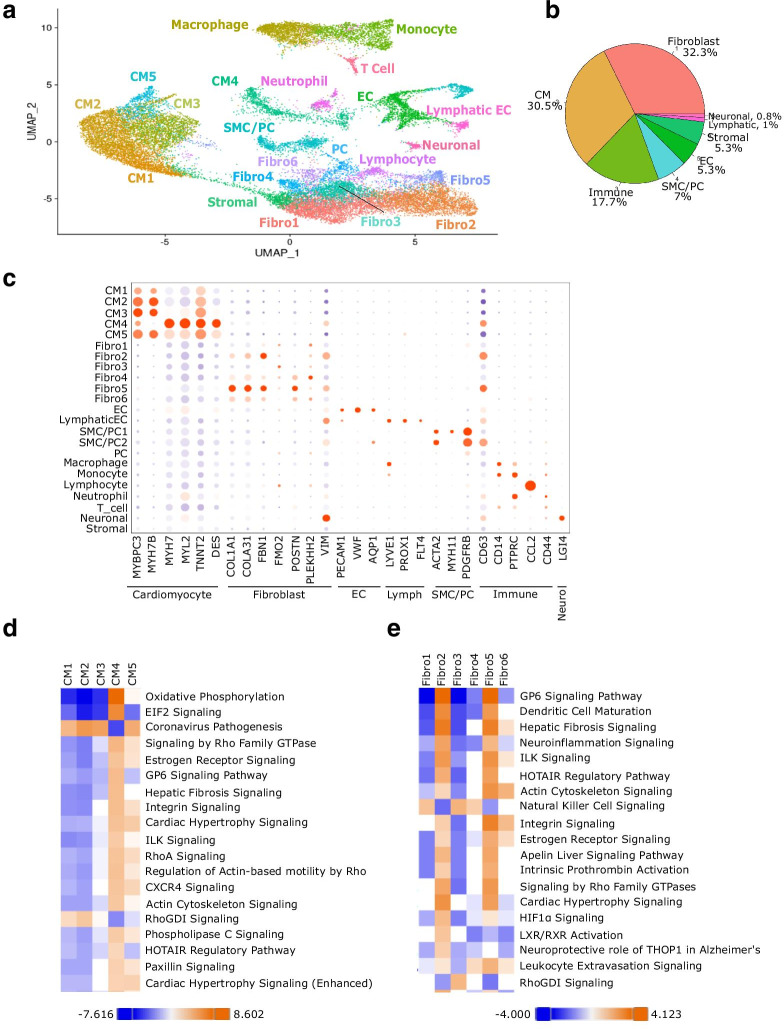
Single nuclei RNA-sequencing reveals cellular diversity within the adult human IVS. **a** UMAP of the integrated dataset overlayed with cell type assignment for each cluster. **b** Distribution of cell types in the adult human IVS. Similar cell types were grouped together. **c** Dotplot of different biomarker expression across all the cell types. The size of the dot indicates the percentage of cells within that cluster expressing that gene, and the color indicates the normalized level of expression. **d** Qiagen Ingenuity Pathway comparison heatmap of the top canonical pathways across the five cardiomyocyte populations within the IVS. **e** Qiagen Ingenuity Pathway comparison heatmap of the top canonical pathways across the six fibroblast populations within the IVS

Cell identities were assigned to each cluster using known biomarkers of expected cell types, differentially expressed genes, gene ontology, and pathway analysis. Similar cell types were positioned close each other on the UMAP (Fig. 4a). The markers used to identify the different cell types are listed in the dot plot (Fig. 4c), and this plot illustrates that each cluster has a unique expression of those markers. Interestingly, we see five separate cardiomyocyte (CM) populations, revealing CM diversity (Fig. 4c, d). Pathway and gene ontology analysis indicate differences in oxidative phosphorylation, protein synthesis, while biomarker analysis reveals differences in expression of sarcomeric proteins such MYH7 and MYH7B. The fourth CM population shows an elevated metabolic phenotype compared to the other four CM populations.

Non-cardiomyocyte cells account for roughly two-thirds of the cells in the human IVS (Fig. 4b), with fibroblasts making up one-third of the total IVS cell population. There are six different fibroblast populations present with considerable differences in gene expression (Fig. 4c). Fibroblast 5 has the greatest expression of collagens, while Fibroblast 1 and 3 show little collagen expression. Ingenuity Pathway Analysis [14] for each fibroblast population illustrates the similarities in biological function for some of these fibroblast populations, such as 1 and 2, and 2 and 5 (Fig. 4e). Interestingly, populations 2 and 5 both exhibit increased activation of proinflammatory and other signaling pathways, indicating an important role in intercellular communication.

Other cell types identified include endothelial, lymphatic, smooth muscle and pericytes, neuronal, and several immune cell populations. Altogether, immune cells account for almost one-fifth of the cells in the adult human IVS (Fig. 4b). Several of the non-myocyte populations were assigned based on gene ontology and pathway analysis. Interestingly, one cell population showed very few upregulated genes, and gene ontology and pathway analysis were not informative. This cell population was labeled as stromal cells, and further analysis of this interesting cell population to understand the role it plays in the normal adult human heart is ongoing.

## Discussion

Biobanking of human heart tissue for advanced molecular biology studies such as single cell genomics has not been standardized. Current published protocols utilize OCT embedding and cryosectioning prior to nuclei isolation for single nuclei RNA-seq [7] or snap freezing of tissue with later mechanical disruption [8]. The former technique is laborious and requires specialized equipment, and the latter technique, while simple, does not include cryopreservative and if not done rapidly enough, tissue is susceptible to ice crystal formation which is expected to disrupt cell and tissue architecture and may reduce the recovery of intact nuclei. Our method, involving simple tissue mincing, addition of a commercially available cryopreservation solution containing dimethyl sulfoxide to limit crystal formation and the use of controlled freezing conditions using commercially available equipment may provide a standard for future biobanking of human heart tissue and is easily accessible to most laboratories without requiring specialized equipment. We demonstrate its utility by generating single nuclei RNA-seq datasets from the IVS of normal human adult hearts.

The IVS is an important and unique anatomical region of the heart, comprising cells of diverse embryological origins, which might explain the cardiomyocyte and fibroblast diversity revealed in this dataset. Alternatively, the diverse cell type subpopulations may also reflect important functional or anatomical niches for each type of cardiomyocyte or fibroblast. Future work applying spatial transcriptomics may provide additional insight.

Our method to cryopreserve and analyze normal human IVS tissue can be applied to normal and diseased human heart tissue from the other regions of the heart and can be done with basic laboratory equipment without requiring extensive specialized expertise or tissue processing, up to the use of commercial single cell microfluidic platforms. Our reference dataset will provide an invaluable resource for comparison of diseased tissue affecting the IVS, as is found in conditions such as hypertrophic cardiomyopathy [15] or ventricular septal defect. It will also complement existing normal human heart single nuclei datasets of the heart [7, 8]. The cell types that we have identified in the interventricular septum correlate with published cell types identified in human ventricular tissue, except that our IVS dataset does not identify adipocytes, and the published ventricular datasets show fewer cardiomyocyte and fibroblast subtypes. Our dataset revealed 8 major cell types present in the IVS, with 23 total distinct cell types within this one region of the heart. Existing human heart single nuclei studies have shown comparable diversity with 9 major cell types and 20 distinct cell types [7], but this was across all four chambers of the heart. Our dataset and theirs show comparable cardiomyocyte and fibroblast diversity. Our analysis also facilitates the distinction between lymphatic and other endothelial cells, between monocytes and macrophages and also between T cells and other lymphocytes, without need for enrichment of these cell populations. Our data also indicates a higher proportion of noncardiac cells within the interventricular septum than the other studies. Reasons for these discrepancies are not clear and possibly result from anatomic divergence, diversity in organ donors, differences in embryological origin of component cells or technical differences in methodology. A true side by side comparison between methods has yet to be performed. Nevertheless, our work will facilitate the identification of abnormal cell types and pathological pathways in diseased human heart tissue, ultimately leading to new targets for therapeutics to treat heart disease.

## Conclusions

We have developed a simple protocol for preserving human heart tissue that can be performed rapidly and simply using commercially available reagents and common laboratory equipment that facilitates analysis by single nuclei transcriptomics. This protocol can be done at varying scales by small and large groups without the need for large consortia, specialized equipment or expertise and will allow scientists with limited resources to biobank and analyze tissue. We demonstrate how this protocol allows the generation of single nuclei transcriptomic data from the normal human heart interventricular septum that is comparable to existing normal human heart datasets generated by much larger consortia and may even capture more complexity. This protocol has the potential to facilitate broader usage of single cell genomics in human heart applications and thus provide greater insights into human heart function, human heart disease and development of therapeutic applications.

## Supplementary Information

**Additional file 1. Supplemental Table 1**. Complete listing of differentially expressed genes in each cluster.

## Data Availability

Single nuclei RNA-seq data were uploaded to the National Center for Biotechnology Information Gene Expression Omnibus database under accession number GSE161921.

## Abbreviations

scRNA-seq: Single cell RNA-sequencing
snRNA-seq: Single nucleus RNA-sequencing
OCT: Optimal cutting temperature compound used for embedding tissue for frozen sectioning
IVS: Interventricular septum
RNA: Ribonucleic acid
RIN: RNA integrity number
QC: Quality control
UMAP: Uniform manifold approximation and projection
CM: Cardiomyocyte
Fibro: Fibroblast
EC: Endothelial cell
SMC: Smooth muscle cell
PC: Pericyte
